# Does the design of the NHS Diabetes Prevention Programme intervention have fidelity to the programme specification? A document analysis

**DOI:** 10.1111/dme.14201

**Published:** 2020-01-03

**Authors:** R. E. Hawkes, E. Cameron, P. Bower, D. P. French

**Affiliations:** ^1^ Division of Psychology and Mental Health Manchester Centre of Health Psychology Manchester UK; ^2^ Division of Population Health Health Services Research & Primary Care University of Manchester Manchester UK; ^3^ Division of Psychology University of Stirling Stirling UK

## Abstract

**Aims:**

To assess fidelity of the Healthier You: NHS Diabetes Prevention Programme (NHS‐DPP), a behavioural intervention for people in England at high risk of developing type 2 diabetes, to the specified programme features.

**Methods:**

Document analysis of the NHS‐DPP programme specification, including National Institute for Health and Care Excellence (NICE) PH38 diabetes prevention guidance. This was compared with the intervention design (framework response documents and programme manuals) from all four independent providers delivering the NHS‐DPP. Documents were coded using the Template for Intervention Description and Replication framework (describing service parameters) and the Behaviour Change Technique Taxonomy v1.

**Results:**

Providers demonstrated good fidelity to service parameters of the NHS‐DPP. The NHS‐DPP specification indicated 19 unique behaviour change techniques. Framework responses for the four providers contained between 24 and 32 distinct behaviour change techniques, and programme manuals contained between 23 and 45 distinct behaviour change techniques, indicating variation in behaviour change content between providers’ intervention documents. Thus, each provider planned to deliver 74% of the unique behaviour change techniques specified, and a large amount of behaviour change content not mandated.

**Conclusions:**

There is good fidelity to the specified service parameters of the NHS‐DPP; however, the four providers planned to deliver approximately three‐quarters of behaviour change techniques specified by the NHS‐DPP. Given that behaviour change techniques are the ‘active ingredients’ of interventions, and some of these techniques in the programme manuals may be missed in practice, this highlights possible limitations with fidelity to the NHS‐DPP programme specification at the intervention design stage.

## What's new?

- The National Health Service (NHS) Diabetes Prevention Programme (NHS‐DPP) is a behavioural intervention for adults in England at risk of developing type 2 diabetes.
- This is the first fidelity evaluation and first known assessment of the behaviour change technique content of a national intervention.
- The NHS‐DPP intervention design demonstrated good fidelity to the service parameters itemized in the programme specification, but programme manuals included only 74% of the specified behaviour change techniques.
- As behaviour change techniques are active ingredients that produce behaviour change, this highlights possible limitations with fidelity to the NHS‐DPP programme specification and dilution of likely effect in NHS‐DPP delivery.

**What's new?**

- The National Health Service (NHS) Diabetes Prevention Programme (NHS‐DPP) is a behavioural intervention for adults in England at risk of developing type 2 diabetes.
- This is the first fidelity evaluation and first known assessment of the behaviour change technique content of a national intervention.
- The NHS‐DPP intervention design demonstrated good fidelity to the service parameters itemized in the programme specification, but programme manuals included only 74% of the specified behaviour change techniques.
- As behaviour change techniques are active ingredients that produce behaviour change, this highlights possible limitations with fidelity to the NHS‐DPP programme specification and dilution of likely effect in NHS‐DPP delivery.

## Introduction

The Healthier You: NHS Diabetes Prevention Programme (NHS‐DPP) is a behavioural intervention focused on encouraging lifestyle behaviour change for adults in England with elevated blood glucose levels 1. The programme was launched in 2016 by NHS England, delivered by four independent provider organizations outside the National Health Service (NHS) who each secured contracts to deliver the programme in localities across England.

The required NHS‐DPP intervention content is outlined within a published Service Specification 2, a framework describing the intervention features which should be present within the NHS‐DPP, informed by reviews of the evidence for lifestyle interventions in the prevention of type 2 diabetes 3, 4. Based on this evidence, the Service Specification requires the NHS‐DPP to be delivered face‐to‐face in groups of no more than 15–20 adults with non‐diabetic hyperglycaemia, over at least 13 sessions (each session lasting 1–2 h, totalling at least 16 h) with a minimum duration of 9 months, in line with existing National Institute for Health and Care Excellence (NICE) guidance on type 2 diabetes prevention 3. The core goals of the intervention are weight loss, improved nutrition and increased physical activity, with use of behaviour change techniques in intervention delivery 2. Behaviour change techniques are defined as observable components of interventions designed to change behaviour, known as ‘active ingredients’ (e.g. goal setting, feedback and self‐monitoring) 5. The included behaviour change techniques have been associated with increased effectiveness in behavioural interventions 6 and diabetes prevention programmes 4.

Given that the Service Specification 2 provides a relatively flexible framework for the NHS‐DPP structure and content, we do not know the final behaviour change technique content of the NHS‐DPP and how this differs between the four provider organizations. Moreover, it is necessary to assess the extent to which the NHS‐DPP is delivered with fidelity to the evidence base in order to draw accurate conclusions about reasons for effectiveness. Intervention fidelity describes whether an intervention is ‘delivered as intended’ 7. If adherence to the intervention design is not evaluated it cannot be ascertained whether intervention effectiveness, or lack thereof, is an intrinsic feature of the NHS‐DPP or due to the intervention not being implemented as intended. The National Institutes of Health Behaviour Change Consortium (NIH‐BCC) model defines five domains of assessing treatment fidelity, including: study design (whether the intervention adequately reflects the evidence base); provider training (whether deliverers are trained in essential components of the intervention); treatment delivery (the extent to which the intervention is delivered with adherence to the protocol); treatment receipt (the extent to which service users understand the intervention); and treatment enactment (whether service users apply what has been learned to their day‐to‐day lives) 7. In line with this guidance, the current manuscript focuses on the first domain in the treatment fidelity model, evaluating fidelity of the NHS‐DPP design.

An evaluation of the NHS‐DPP at the formative stage 8 highlighted the need for fidelity measures to be established. A process evaluation of the demonstrator phase and first wave roll‐out of the NHS‐DPP in 2015 and 2016, respectively, reported good fidelity to the intervention design 9, however, limitations of this evaluation are evident. These prior analyses had access to providers’ framework response documentation but not to the programme manuals. The framework responses were submitted by each provider during bids for service procurement, detailing the overall proposed structure and content of their interventions. Programme manuals were developed after providers had secured service provision, describing a session‐by‐session protocol for NHS‐DPP delivery. The behaviour change technique content described in the programme manuals is therefore more proximal to providers’ NHS‐DPP intervention plans.

This prior evaluation 9 used a reduced and non‐standardized coding frame for assessing behaviour change techniques. For example, the most commonly used taxonomy, the Behaviour Change Technique Taxonomy (BCTTv1) 5, lists two separate techniques for setting behavioural and outcome goals, but these were reduced to one technique labelled ‘goal setting’. However, inclusion of both behavioural goals and outcome goals was recommended by the programme specification 2, 3 as these require different types of goals to be set (e.g. to increase physical activity and reduce weight), and it is important to know exactly which of these is included in the intervention. This article includes the providers’ programme manuals in addition to the framework response documents and uses the full version of the BCTTv1.

Previous reviews have focused mostly on fidelity of intervention delivery (e.g. English Stop‐Smoking Services 10). This article extends the current literature by providing the first known evaluation of fidelity of design of a national multisite intervention. This method builds on NIH‐BCC suggestions for measuring fidelity of design 11. The objectives of this document analysis were to describe the content and techniques of the NHS‐DPP, examine variation in NHS‐DPP designs between providers, and determine whether the NHS‐DPP intervention has been designed with fidelity to the programme specification 2, 3. The term ‘providers’ refers to the four commercial companies commissioned to deliver the NHS‐DPP.

## Methods

### Document review

A comparison was made of the programme specification and the intervention design. The full programme specification (describing what should be present within the NHS‐DPP) consisted of:

- NHS England NHS‐DPP Service Specification (v.01, March 2016) 2, specific to the commissioning of the NHS‐DPP, which draws on recommendations from NICE PH38 3;
- NICE PH38 public health guideline 3, ‘Type 2 diabetes: prevention in people at high risk’, providing general guidance on what diabetes prevention programmes should look like and describing additional information regarding behaviour change content.

The intervention design (describing what providers plan to deliver) consisted of:

- four framework response documents (one per provider organization) describing the proposed service delivery, submitted in providers’ bids for service procurement;
- six programme manuals (one each for two provider organizations, and two each for the other two providers).

### Coding frameworks

The 12 documents were examined using two frameworks. Planned programme content for the full programme specification and each of the providers’ intervention designs was reviewed using the Template for Intervention Description and Replication (TIDieR) framework 12; a data extraction tool in which the materials, procedures, mode of delivery, location, dose and tailoring for the NHS‐DPP were documented. The intended behaviour change technique content identified in each of the 12 documents was coded using the BCTTv1 5, defining 93 distinct techniques. Both frameworks have been used widely for reporting and evaluating interventions, with the BCTTv1 evidencing good intercoder reliability, test–retest reliability and good validity 13.

### Coding procedures

For each of the 12 documents reviewed, data extraction and coding was carried out independently by two researchers (EC and RH). TIDieR variables were extracted and behaviour change techniques were coded using author‐developed data collection forms (see Doc. S1 detailing coding procedures). Researchers underwent training in the use of the BCTTv1 14. A set of coding rules was developed through team discussions, all documents were coded separately following guidance from taxonomy authors. Coding rules stated that new behaviour change techniques were to be coded on commencement of a new activity or if a different health behaviour (e.g. diet, physical activity) was targeted. The level of target behaviour was also documented when coding the technique ‘information about health consequences’ (e.g. levels of the target behaviour ‘diet’ included information about carbohydrates, fats, sugar, etc.) as coders felt these were distinct pieces of information targeting distinct behaviours.

### Analysis

Interrater reliability (IRR) was calculated using Cohen's kappa coefficient 15 to determine consistency between coders for use of the BCTTv1. IRR values were determined for the NHS Service Specification and NICE PH38 guideline, each provider framework response, and for each session within each of the provider manuals. Identified coding discrepancies were discussed between EC, RH and DF until agreement was met and a final set of behaviour change techniques was determined for each document. The numbers of different techniques in each document were calculated and labels of these techniques were recorded. Behaviour change techniques present in the Service Specification and NICE guidance (programme specification) were compared with those present in framework response documents and programme manuals (providers’ intervention designs).

### Ethics statement

This study analysed only written documents and did not include data collected from human participants, and therefore falls outside the remit of NHS Research Ethics Committees. However, the wider programme of research of which this study is a part was reviewed and approved by the North West Greater Manchester East NHS Research Ethics Committee (ref. 17/NW/0426, 1 August 2017).

## Results

### Service parameters

The service parameters specified by NHS England 2 and NICE PH38 guidance 3 in comparison with each provider intervention design are shown in Table 1 (extracted using the TIDieR framework) 12. All four providers generally had good fidelity of design for duration and frequency of sessions. Providers 1 and 3 stated plans in their framework responses to deliver groups with a maximum of 20 people; this corresponds with requirement from the NHS Specification, but is more than the recommended 10–15 service users per group recommended by NICE.

**Table 1:** Service parameters outlined in the programme specification in comparison with each provider intervention design

	NICE PH38	NHS specification		Provider		
			1	2	3	4
Group size	10–15	Maximum 20	Maximum 20	10–15	15–20	Maximum 15
No. of sessions	Minimum 8	Minimum 13	18	13	10 (+3 review sessions)	13
Contact time	Minimum 16 h	Minimum 16 h	18.5 h	16 h	13 h (+3)	19.5 h
Duration	9–18 months with follow‐up sessions for 2 years	Minimum 9 months	9 months	12 months	9 months	10 months
Frequency	Tapered	Allow sufficient time between sessions to make gradual behaviour changes	6 weekly sessions, followed by 6 fortnightly sessions, followed by 6 monthly sessions	8 weekly sessions, and follow‐up sessions at 2, 3, 4.5, 6, 9 and 12 months	6 fortnightly closed group sessions and 4 monthly open group maintenance sessions. One‐to‐one reviews at 3, 6 and 9 months	4 weekly closed group sessions, followed by 9 monthly semi‐open sessions
Session duration	–	1–2 h	1 hour (first session 1.5 h)	1.5 h core sessions (first session 1 h), 1 h follow‐up sessions	1.5 h core sessions, 1 h maintenance sessions, 1 h review sessions	1.5 h
Mode of delivery	Individual or group sessions	Face‐to‐face group sessions	Face‐to‐face group sessions	Face‐to‐face group sessions	Face‐to‐face group sessions	Face‐to‐face group sessions
Weigh‐ins	–	Every session	Every session	Every session	Every session	Every session

Note

Providers 1, 2, 3 and 4 in Table 1 do not correspond to providers A, B, C and D in Tables 2 and 3 to preserve anonymity for provider organizations.

NICE, National Institute for Health and Care Excellence; NHS, National Health Service.

### Behaviour change technique content

The intended behaviour change technique content from the full programme specification (NHS Service Specification 2 and NICE guidance 3) in comparison with the planned behaviour change content from the framework responses of each provider is shown in Table 2. The intended behaviour change content from the full programme specification compared with the planned content from each of the providers’ programme manuals is shown in Table 3.

**Table 2:**
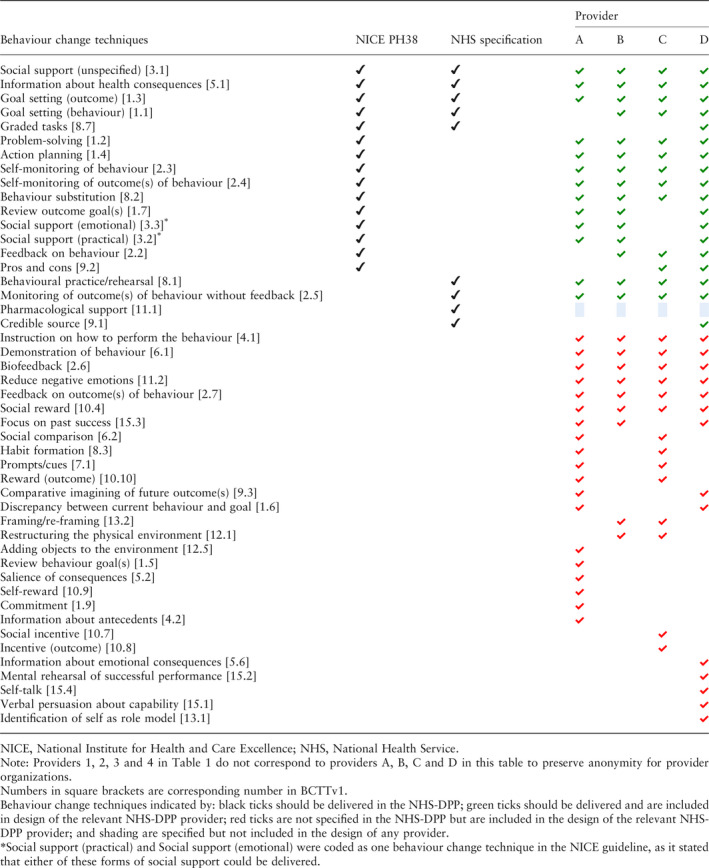
Behaviour change techniques specified in the programme specification compared with behaviour change techniques specified in providers’ framework response documents

**Table 3:**
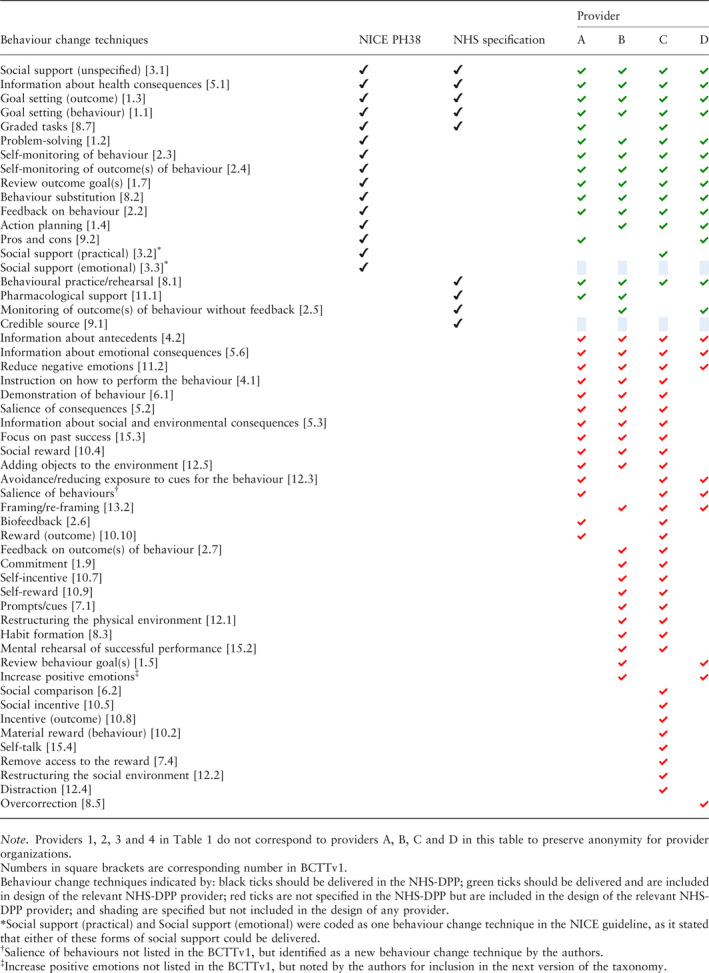
Behaviour change techniques specified in the programme specification compared with behaviour change techniques specified in providers’ programme manuals

Kappa values ranged from 0.75 to 0.88 for each of the documents, demonstrating high agreement, prior to resolving discrepancies in coding (see Table S1 displaying IRR values 15).

#### Programme specification

In total, 19 unique behaviour change techniques were coded in the full programme specification; nine were identified in the NHS Service Specification 2 and 15 in the NICE guideline 3 (which provided more detailed guidance on behaviour change technique content). Five techniques were present in both documents, including goal setting for behaviour, goal setting for health outcomes, unspecified social support, information about health consequences and graded tasks. Table 4 briefly defines the 19 specified behaviour change techniques.

**Table 4:** Behaviour change technique definitions

Behaviour change technique	Definition
Unspecified social support [3.1]	Advise on, arrange or provide social support or non‐contingent praise or reward for performance of the behaviour
Information about health consequences [5.1]	Provide information about health consequences of performing the behaviour
Goal setting for health outcomes [1.3]	Set or agree on a goal defined in terms of a positive outcome of wanted behaviour
Goal setting for health behaviours [1.1]	Set or agree on a goal defined in terms of the behaviour to be achieved
Graded tasks [8.7]	Set easy‐to‐perform tasks, making them increasingly difficult, but achievable, until behaviour is performed
Problem‐solving [1.2]	Prompt the person to analyse factors influencing the behaviour and generate or select strategies that include overcoming barriers or increasing facilitators
Self‐monitoring of behaviour [2.3]	Establish a method for the person to monitor and record their behaviour(s) as part of a behaviour change strategy
Self‐monitoring of outcomes of behaviour [2.4]	Establish a method for the person to monitor and record the outcome(s) their behaviour as part of a behaviour change strategy
Reviewing outcome goals [1.7]	Review outcome goal(s) jointly with the person and consider modifying goal(s) in light of achievement
Behaviour substitution [8.2]	Prompt the substitution of the unwanted behaviour with a wanted or neutral behaviour
Giving feedback on behaviour [2.2]	Monitor and provide informative or evaluative feedback on performance of the behaviour
Action planning [1.4]	Prompt detailed planning of the performance of the behaviour (must include at least one of context, frequency, duration and intensity).
Pros and cons [9.2]	Advise the person to identify and compare reasons for wanting (pros) and not wanting to (cons) change the behaviour
Practical social support [3.2]	Advise on, arrange or provide practical help for performance of the behaviour
Emotional social support [3.3]	Advise on, arrange or provide emotional social support for performance of the behaviour
Behavioural practice [8.1]	Prompt practice or rehearsal of the performance of the behaviour in order to increase habit or skill
Pharmacological support [11.1]	Provide, or encourage the use of or adherence to, drugs to facilitate behaviour change
Monitoring outcome of behaviour by others without feedback [2.5]	Observe or record outcomes of behaviour with the person's knowledge as part of a behaviour change strategy
Credible source [9.1]	Present verbal or visual communication from a credible source in favour of or against the behaviour

Definitions are summarized from BCTTv1. Numbers in square brackets correspond to numbers in BCTTv1.

#### Intervention design

According to the framework response documents, providers A, B, C and D stated plans to deliver 32, 24, 27 and 32 unique behaviour change techniques respectively (see Table 2). The most commonly cited technique in each framework response was unspecified social support. In the programme manuals, a total of 28, 35, 45 and 23 unique behaviour change techniques were identified for providers A, B, C and D respectively (see Table 3). The most common technique in each programme manual was giving information about health consequences.

There was a difference in the number and type of behaviour change techniques stated within provider's own framework responses and programme manuals. Providers B and C stated plans to deliver more techniques according to their programme manuals (11 and 18 additional techniques respectively) compared with their framework responses. Providers A and D stated plans to deliver more techniques according to their framework responses (four and nine additional techniques respectively), which did not track through to their programme manuals. Given that providers’ manuals are more proximal to the planned delivery, this suggests dilution in planned behaviour change content. Across the programme manuals and framework responses combined, a total of 41, 38, 47 and 39 behaviour change techniques were identified in providers’ intervention designs respectively.

### Fidelity evaluation: behaviour change technique content

Behaviour change technique content was compared between the full programme specification and providers’ intervention designs to assess fidelity of the NHS‐DPP design. Of the 19 specified behaviour change techniques in the full programme specification, providers included 13 (68%), 15 (79%), 13 (68%) and 18 (95%) specified techniques respectively in their framework responses (see Table 2). In their programme manuals, all four providers included 14 (74%) of these behaviour change techniques (see Table 3), however, these were not the same 14 techniques. The techniques ‘providing emotional social support’ and ‘credible source’ were not mentioned in any of the providers’ programme manuals, although emotional social support was mentioned in three providers’ framework responses. Eleven of the techniques cited in the full programme specification were included in all four providers’ manuals. Eight techniques were not described by at least one provider, indicating a lack of fidelity to the programme specification by behaviour change technique omission. There were 34 techniques included in at least one provider's programme manual that had not been specified by the NHS Service Specification 2 or NICE PH38 guideline 3, indicating a lack of fidelity to the programme specification by behaviour change technique addition.

## Discussion

Providers generally demonstrated good fidelity to the service parameters of the NHS‐DPP design, but according to their programme manuals only planned to deliver 14 of the 19 (74%) behaviour change techniques specified by NHS England 2 and NICE 3. The 14 techniques varied across the four providers, indicating variation in the ‘active ingredients’ within providers’ intervention designs. Sixty‐four per cent of planned techniques across all of the programme manuals were not specified by NHS England or NICE.

### Strengths and limitations

The current analysis provides a detailed understanding of the whole NHS‐DPP intervention design; it utilized more recent documentation compared with a previous fidelity analysis of the NHS‐DPP 9 and included use of the full BCTTv1 5. The authors used standardized tools 5, 12 and obtained all relevant documentation to complete the analysis. It should be noted that framework response documents were submitted by providers during early stages of service procurement, and may have since changed some aspects of intervention characteristics. Framework responses described important information regarding providers’ service delivery plans (extracted using the TIDieR framework) 12, and contained potential behaviour change content intended to be present across the whole intervention. Together with the programme manuals, this gave a comprehensive understanding of the proposed NHS‐DPP intervention design.

All techniques that could result in behaviour change, according to the BCTTv1 5, were identified in providers’ documentation. However, despite identifying all behaviour change strategies, we cannot ascertain what exactly the providers *intended* to be the key ‘active ingredients’ in their intervention designs. Similarly, authors coded all identifiable techniques in the full programme specification that were recognized as being required by NHS England, and thereby determined the ‘active ingredients’ of the programme specification. It is noted that the NHS Service Specification 2 and NICE guideline 3 offer some differing recommendations on what intervention features should be present in the NHS‐DPP (Table 1). These differences in the programme specification documents could be one reason for the variability in providers’ framework responses and programme manuals.

This article provides a reliable method for assessing fidelity of intervention design using behaviour change technique coding; kappa values demonstrated high agreement between coders. However, providers were considered to demonstrate fidelity when a behaviour change technique stated in the full programme specification was present in providers’ intervention design documents, although there is no compelling evidence that use of a technique once is sufficient.

Finally, Hawe and colleagues 16 argued that standardizing complex intervention designs fails to capture the essence of the intervention; rather than standardizing individual intervention components, the key processes of change (i.e. the functions) should be standardized to enable interventions to be tailored according to context. However, in our opinion, the specified techniques in the NHS‐DPP intervention design are the function of the NHS‐DPP because they are the ‘active ingredients’ that may produce behaviour change, according to the evidence base 2, 3, 4.

### Relation to existing research

Providers generally demonstrated good fidelity to the service parameters of the NHS‐DPP design, but according to their programme manuals only planned to deliver 74% of the specified behaviour change techniques in the programme specification 2, 3. There is no criteria for what is considered ‘good’ fidelity, and to the authors’ knowledge there is no research evaluating fidelity of design of another comparable programme. However, there is a general consensus that > 80% demonstrates ‘high’ fidelity and < 50% demonstrates ‘low’ fidelity 10, 11. Some findings are consistent with the previous fidelity assessment of the NHS‐DPP demonstrator phase 9 ^,^ which reported good fidelity of the intervention design. However, the current analysis has identified a 26% loss of fidelity by behaviour change technique omission according to providers’ programme manuals. The programme manuals describe a session‐by‐session protocol for delivering the NHS‐DPP in practice, therefore likely providing the most accurate representation of the planned techniques in each providers’ intervention design. Programme manuals were not available for the previous fidelity assessment of the NHS‐DPP 9.

This is the first known assessment of behaviour change technique content of a national diabetes prevention programme intervention and one of the first fidelity evaluations of a national intervention. Previous diabetes prevention trials 17, 18, 19, 20, 21 have not provided a robust evaluation of fidelity, nor have they described or evaluated behaviour change techniques used in intervention designs. The current analysis will allow for more accurate conclusions to be drawn about reasons for effectiveness (or ineffectiveness) of the NHS‐DPP and has identified the ‘active ingredients’ included in intervention design. The effectiveness of lifestyle change in the prevention of type 2 diabetes has been demonstrated in randomized controlled trials in Finland 17 and the USA 18, both of which reported a 58% relative risk reduction for those who undertook lifestyle interventions. These findings have also been replicated in Chinese 19, Japanese 20 and Indian 21 populations. However, translating these programmes into practice has remained a challenge, and benefit is likely to be lower in routine implementation. As only 74% of behaviour change techniques are specified in providers’ intervention protocols, there is likely to be further dilution of fidelity in the delivery of behaviour change techniques in the field; this will be assessed in the next stage of our fidelity analyses.

### Implications for practice

Sixty‐four per cent of planned behaviour change techniques across all of the programme manuals were not specified by NHS England or NICE. However, we do not yet know whether this is problematic as the NHS‐DPP is evidence‐inspired rather than evidence‐based. That is, there is not yet any effectiveness analysis of the NHS‐DPP, only expert opinion guided by evidence from related research 22.

The impact of both absent and additional non‐specified behaviour change techniques on the effectiveness of the NHS‐DPP is yet to be established. Previous systematic reviews suggest the provision of social support and ‘self‐regulatory’ techniques such as goal‐setting, self‐monitoring and feedback are effective in interventions targeting diet, physical activity and weight loss 6, 23, 24, 25, 26, 27, and goal‐setting techniques are effective in type 2 diabetes self‐management interventions 28. Some of the 34 non‐specified techniques present in providers’ programme manuals are self‐regulatory or have at least some evidence of effectiveness in interventions with similar populations or target behaviours as the NHS‐DPP. For example, the techniques ‘instruction on how to perform a behaviour’ and ‘demonstration of the behaviour’ have been associated with a significant reduction in HbA_1c_ in people with type 2 diabetes 29. There is little evidence regarding effectiveness of the other non‐specified behaviour change techniques. It is possible that interventions containing more strategies to help people change their diet and physical activity behaviours may be more effective 23. However, this variance between providers’ intervention designs reduces the consistency of the planned intervention content of the NHS‐DPP.

Already as a result of this document analysis, NHS England have stated requirements for providers to be more explicit regarding behaviour change strategies in their framework response documents which track through to their programme manuals; this requirement is stated in the new NHS Service Specification 2 for subsequent phases of the NHS‐DPP intervention roll‐out.

### Implications for research

This article provides a method for measuring fidelity of a national programme's intervention design, extending on previous fidelity research which, to date, has focused more on evaluating fidelity of intervention delivery. This method builds on suggestions set out by the NIH‐BCC 11.

The present research has identified all key behaviour change techniques in the NHS‐DPP intervention design, providing a baseline description for evaluating later steps in the NIH‐BCC model 7; ongoing research will examine the training (observations of NHS‐DPP staff training sessions), delivery (observations of NHS‐DPP course delivery) and receipt (interviews with service users about their experience of the course). Future research should also consider what is a sufficient ‘dose’ of behaviour change techniques to produce behaviour change, and to also establish what levels of fidelity of national programmes with multiple providers produce and how this impacts on effectiveness of those programmes.

## Conclusions

The current document analysis of the NHS‐DPP intervention design has identified generally good fidelity to the service parameters of the NHS‐DPP programme specification. However, according to their programme manuals, providers planned to deliver only 74% of behaviour change techniques specified by NHS England 2 and NICE 3, and a large amount of additional behaviour change content not mandated. This means the NHS‐DPP intervention design has a 26% loss of fidelity by behaviour change technique omission. Given that behaviour change techniques are the ‘active ingredients’ of an intervention, and some techniques in the programme manuals may be missed in practice, this highlights a possible lack of fidelity to the NHS‐DPP programme specification at the intervention design stage.

## Funding sources

This work is independent research funded by the National Institute for Health Research [Health Services and Delivery Research, 16/48/07 – Evaluating the NHS Diabetes Prevention Programme (NHS DPP): the DIPLOMA research programme (Diabetes Prevention – Long Term Multimethod Assessment)]. The views and opinions expressed in this manuscript are those of the authors and do not necessarily reflect those of the National Institute for Health Research or the Department of Health and Social Care.

## Competing interests

None declared.

## Supporting information


**Table S1.** Cohen's kappa values for each specification document and provider document.
**Doc. S1.** Behaviour change technique coding procedures.
