# Supplementary material for: Does the design of the NHS Diabetes Prevention Programme intervention have fidelity to the programme specification? A document analysis
Source: Diabet Med. 2020 Jan 3;37(8):1357–66. doi: 10.1111/dme.14201 (PMC7496858; doi:10.1111/dme.14201)
Supplement: Supplementary file 1 — Table S1. Cohen's kappa values for each specification document and provider document. Doc. S1. Behaviour change technique coding procedures. [file DME-37-1357-s001.docx]

**Does the design of the NHS Diabetes Prevention Programme intervention have fidelity to the programme specification? A document analysis**

R. E. Hawkes, E. Cameron, P. Bower and D. P. French

**Doc. S1. Behaviour change technique coding procedures**

Behaviour change techniques were coded using an author developed table, which included behaviour change technique name, confidence of presence, information about behavioural targets (e.g. diet, physical activity), mode of delivery (e.g. visual, auditory, text), group or individual delivery of behaviour change technique, whether the behaviour change technique was delivered interactively or passively (i.e. whether the behaviour change technique required an action from the service users), and whether it was optional or required of the NHS-DPP deliverers.

Coding rules stated that new behaviour change techniques would be coded on the commencement of a new activity or if a different health behaviour (e.g. diet, physical activity) was targeted. The level of target behaviour was also documented when coding the behaviour change technique ‘information about health consequences’ (e.g., levels of the target behaviour ‘diet’ included information about carbohydrates, fats, sugar, etc.) as the authors felt these were distinct pieces of information targeting distinct behaviours. A coding rule established though team discussions instructed to code as ‘information about health consequences’ when interrupted by other activities that did not comprise of information about health consequences, or when a new ‘level’ of behaviour was targeted (e.g. information provided about the health consequences of carbohydrates followed by information about the health consequences of fruit and vegetables). Consequently, this meant the behaviour change technique ‘information about health consequences’ was coded more frequently than others.

Behaviour change techniques present in both the full programme specification and the providers’ intervention designs were documented as indicating fidelity to the programme specification. Behaviour change technique stated in the full programme specification that were not present in the intervention designs, and additional behaviour change techniques identified in the programme manuals and framework response documents which were not otherwise specified, were documented as indicating non-fidelity to the programme specification.

**Table S1. Cohen’s Kappa values for each specification document and provider document**

| **Source Document** | **Kappa Value** |
| --- | --- |
| NHS Service Specification | **0.839** |
| NICE PH38 guidelines | **0.870** |
| **Provider 1** | |
| **Framework response** | **0.757** |
| Session 1 | 0.754 |
| Session 2 | 0.730 |
| Session 3 | 0.870 |
| Session 4 | 0.816 |
| Session 5 | 0.784 |
| Session 6 | 0.764 |
| Session 7 | 0.730 |
| Session 8 | 0.832 |
| Session 9 | 0.832 |
| Session 10 | 0.832 |
| Session 11 | 0.640 |
| Session 12 | 0.762 |
| Session 13 | 0.770 |
| Session 14 | 0.784 |
| Session 15 | 0.821 |
| Session 16 | 0.769 |
| Session 17 | 0.713 |
| Session 18 | 0.776 |
| **Overall programme manual** | **0.806** |
| **Provider 2** | |
| **Framework response** | **0.878** |
| Session 1 | 0.935 |
| Session 2 | 0.691 |
| Session 3 | 0.664 |
| Session 4 | 0.631 |
| Session 5 | 0.753 |
| Session 6 | 0.631 |
| Session 7 | 0.654 |
| Session 8 | 0.293^a^ |
| Session 9 | 0.470^a^ |
| Session 10 | 0.332^a^ |
| Session 11 | 0.332^a^ |
| Session 12 | 0.332^a^ |
| Session 13 | 0.332^a^ |
| **Overall programme manual** | **0.754** |
| **Provider 3** | |
| **Framework response** | **0.840** |
| Session 1 | 0.707 |
| Session 2 | 0.732 |
| Session 3 | 0.776 |
| Session 4 | 0.727 |
| Session 5 | 0.619 |
| Session 6 | 0.946 |
| Session 7 | No docs |
| Session 8 | 0.591 |
| Session 9 | 0.654 |
| Session 10 | 0.655 |
| Session 11 | 0.669 |
| Session 12 | No docs |
| **Overall programme manual** | **0.788** |
| **Provider 4** | |
| **Framework response** | **0.750** |
| Session 1 | 0.789 |
| Session 2 | 0.784 |
| Session 3 | 0.694 |
| Session 4 | 0.708 |
| Session 5 | 0.708 |
| Session 6 | 0.708 |
| Session 7 | 0.709 |
| Session 8 | 0.625 |
| Session 9 | 0.677 |
| Session 10 | 0.777 |
| Session 11 | 0.709 |
| Session 12 | 0.754 |
| Session 13 | 0.555 |
| **Overall programme manual** | **0.763** |

*Note: Providers 1, 2, 3 and 4 does not correspond to providers A, B, C and D in BCT Tables 2 and 3 to preserve anonymity for provider organisations.*

^a^ *Kappa values for the review sessions of provider 2 (sessions 8-13) were low. Provider 2’s manual contained one page to explain six review sessions which led to some differing interpretations between coders due to some ambiguity. Discrepancies were resolved and the overall programme manual demonstrated high agreement between coders across all sessions (kappa = 0.75).*
